# Viral metagenomics updated the prevalence of human papillomavirus types in anogenital warts

**DOI:** 10.1080/22221751.2019.1661757

**Published:** 2019-09-07

**Authors:** Hui Xu, Yu Ling, Yuan Xi, Hong Ma, Hao Wang, Hui-Min Hu, Qi Liu, Yu-Mei Li, Xu-Tao Deng, Shi-Xing Yang, Eric Delwart, Wen Zhang

**Affiliations:** a Department of Dermatology, the Affiliated Hospital of Jiangsu University, Zhenjiang, People’s Republic of China; b Department of Microbiology, School of Medicine, Jiangsu University, Zhenjiang, People’s Republic of China; c Vitalant Research Institute, San Francisco, CA , USA; d Department of Laboratory Medicine, University of California San Francisco, San Francisco, CA , USA

**Keywords:** Human papillomavirus, anogenital wart, viral metagenomics, complete genome, HPV7

## Abstract

To investigate the composition of human papillomavirus (HPV) types in anogenital warts (AGWs), viral nucleic acid in 110 AGWs, pooled into 11 specimen pools, were subjected to viral metagenomic analysis. After finding HPV7 in AGWs, conventional PCR screening was performed for HPV7 in other 190 individual AGW specimens. Viral metagenomic results indicated that 29 different types of HPV were recovered, with HPV11 and HPV6 showing the highest proportion of sequence reads. HPV7 was detected in 7 of 11 pools, 5 of which contained abundant HPV7 sequence reads. 24 complete genomes of HPV were acquired in viral metagenomic analysis, including 5 HPV7 genomes, based on which phylogenetic analysis and pairwise sequence comparison were conducted. PCR screening for HPV7 in other 190 individual AGW specimens revealed 25 positive cases (13.16%), of which the amplified fragments were sequenced and confirmed to be HPV7 sequences. Although HPV7 was generally found in hand warts and recently also in warts in toe webs, our data suggested that the role of HPV7 in AGW should be considered in the future clinical test and vaccine development for AGWs.

## Introduction

Human papillomavirus (HPV) infections are very common and viral DNA can be detected from skin, oral and anogenital samples from all human populations [1]. Thus far, over 200 types of HPV have been fully characterized and predominantly assigned into three genera: *Alphapapillomavirus*, *Betapapillomavirus* and *Gammapapillomavirus* [2,3]. Anogenital wart (AGW) is one of the most widespread sexually transmitted diseases caused by HPV, being generally associated with HPV6 and HPV11 [4,5]. Although many other HPV types including HPV 16, 18, 30–33, 35, 39, 41–45, 51–56, and 59 have also been detected in AGWs [6–8], the currently licensed HPV vaccines for AGW mainly provide protection against infections with HPV6 and HPV11 due to the fact that the two types of HPV accounted for more than 80% of the cases [9]. Rising prevalence and incidence of AGWs in the general population represents a significant HPV disease burden worldwide, resulting in substantial healthcare costs and deteriorating quality of life [10–12]. A systematic review of the epidemiology of AGWs in the literature indicated that the overall (females and males combined) reported an annual incidence of any AGWs (including new and recurrent) ranged from 160 to 289 per 100,000, with a median of 194.5 per 100,000 [10].

Understanding the prevalence of different types of HPV in AGWs will provide an epidemiological basis for developing the most cost-effective strategies to prevent and control HPV infection. Therefore, in this study, using the viral metagenomic technique, we investigated the HPV infection in AGWs in Zhenjiang City in China, which revealed that besides HPV6 and HPV11, HVP7 is also a common type prevalent in AGWs.

## Materials and methods

### Sample collection and preparation

For viral metagenomic analysis, 110 wart specimens were collected from 110 patients (including 76 male and 34 female) with AGWs who were admitted to the Department of Dermatology and Venerology at the Affiliated Hospital of Jiangsu University from September 2016 to August 2017. Detailed information of these patients was included in Table S1. The mean age of study participants was 42.5 ± 15.56 (16–84) years. The average courses were 3.26 ± 3.14 month (1 week to 15 months). The 110 warts were randomly pooled into 11 specimen pools, each including 10 warts. The 11 wart pools were individually homogenized with a mortar and pestle, resuspended in 1 mL DPBS, and then frozen and thawed rapidly three times on dry-ice. The supernatants were then collected after centrifugation (10 min, 15,000× g). Supernatants were filtered through 0.45-mm filters (Millipore) to remove eukaryotic- and bacterial cell-sized particles, and 200 uL of supernatant from each pool was then subjected to a mixture of nuclease enzymes to reduce the concentration of free (non-viral encapsidated) nucleic acids [13,14]. Remaining total nucleic acid was then isolated using QIAamp MinElute Virus Spin Kit according to the manufacturer’s protocol.

For PCR screening for HPV7 in individual AGW specimen, 190 specimens were collected from190 patients (including 146 male and 44 female) with AGWs who were admitted to the Department of Dermatology and Venerology at the Affiliated Hospital of Jiangsu University from August 2017 to April 2019. The mean age of study participants was 40.06 ± 14.08 (18-88) years. The average courses were 3.56 ± 3.00 month (2 weeks to 24 months). The information of these specimens was listed in Table S2. These specimens were divided into two: one part for histopathological examination and the other for viral nucleic acid extraction. For viral nucleic acid extraction, these wart specimens were individually homogenized with a mortar and pestle, resuspended in 0.5 mL DPBS, and then frozen and thawed rapidly three times on dry-ice. The supernatants were then collected after centrifugation (10 min, 15,000× g) and subjected to viral nucleic acid extraction using QIAamp MinElute Virus Spin Kit according to the manufacturer’s protocol.

All patients included in this study were negative for Treponema pallidum, lupus erythematosus, hepatitis B virus, hepatitis C virus and human immunodeficiency virus, and no patients included in this study had severe systemic diseases.

### Viral metagenomic sequencing and bioinformatic analysis

Eleven libraries were then constructed using Nextera XT DNA Sample Preparation Kit (Illumina) and sequenced using the MiSeq Illumina platform with 250 bases paired ends with dual barcoding for each pool. For bioinformatics analysis, paired-end reads of 250 bp generated by MiSeq were de-barcoded using vendor software from Illumina. An in-house analysis pipeline running on a 32-nodes Linux cluster was used to process the data. Low sequencing quality tails were trimmed using Phred quality score ten as the threshold. Adaptors were trimmed using the default parameters of VecScreen which is NCBI BLASTn with specialized parameters designed for adapter removal. The cleaned reads were *de novo* assembled within each barcode using the ENSEMBLE assembler [15]. Contigs and unassembled reads were then matched against a customized viral proteome database using BLASTx with an E-value cutoff of <10^−5^, where the virus BLASTx database was compiled using NCBI virus reference proteome (ftp://ftp.ncbi.nih.gov/refseq/release/viral/) to which was added viral proteins sequences from NCBI nr fasta file (based on annotation taxonomy in Virus Kingdom). To reduce false positives, candidate viral hits were compared to an in-house non-virus non-redundant (NVNR) protein database using DIAMOND [16], where the NVNR database was compiled using non-viral protein sequences extracted from NCBI nr fasta file (based on annotation taxonomy excluding Virus Kingdom). Hits showing a higher (less significant) E score to NVNR than to viral sequences were removed.

### Reads distribution and genome acquisition of HPVs

The BLASTx results were loaded into the MEGAN program for viewing profiles and HPV type attributes [17]. For complete HPV genome acquisition, sequence reads within each barcode were *de novo* assembled into contigs using CLC genomics workbench 10.0 with stringent parameters and resulted contigs were subjected to BLASTx search against the customized HPV proteome database which was compiled using proteins sequences of viruses in *Papillomaviridae* family based on annotation taxonomy in Virus Kingdom. Only the assembled sequences showed at least 50 bp overlapping at the start and end, confirming their circular genomes, were considered to be putative complete HPV genome and subjected to further ORF finding.

### Phylogenetic analysis

Phylogenetic analysis was performed based on the complete genome sequences of the 24 HPVs acquired here, their closest relatives based on BLASTn, and other representative HPV types in genus *Alphapapillomavirus*. Sequence alignment was performed using CLUSTAL W with the default settings [18]. A phylogenetic tree with 1000 bootstrap resamples of the alignment data sets was generated using the maximum-likelihood (ML) method in MEGA7.0. Bootstrap values for each node were given [19].

### PCR screening for HPV7 in individual AGW specimens

To investigate the prevalence of HPV7 in AGWs, two sets of PCR primers were respectively designed based on the conserved sequences in the E1 and L1 gene regions after aligning the five HPV7 genomes identified in this study and the reference HPV7 complete genome sequence available in GenBank (NC_001595). We used nested primers to increase the sensitivity of PCR amplification. The primers based on L1 gene region are L1pwsFO (5′-ACAATCCCAGACGCCTTGAG-3′) and L1pwxRO (5′-CTATGAGGCCTGCAGCAACT-3′) for the 1st round PCR, and L1pnsFI (5′-ACCATACCAAAGACCACACCT-3′) and L1pnxRI (5′-TTCCCACAGTTTACAGGCCC-3′) for the 2nd round PCR. The PCR product size of the 2nd round PCR is 357 bp. The primers based on E1 gene region are E1pwsO (5′- AGTTGACAGAAACAGCTCCATC-3′) and E1pwxRO (5′- GCAGGCAGCAGATGTAGTTT-3′) for the 1st round PCR, and E1pnsFI (5′-TGTGAATTCCTTGTCCCGGC-3′) and E1pnxRI (5′-AGCAGAGAAGAACTTTTGGCAG-3′) for the 2nd round PCR. The PCR product size of the 2nd round PCR is 300 bp. The specific DNA bands were sequenced by Sanger method. Standard precautions were used to prevent the cross-sample contamination.

### Study approval

Patients signed informed consent forms, and protocols were approved by Medical Ethical Committee at the Affiliated Hospital of Jiangsu University (Reference code: ujsah2016219).

## Results

### Viral sequences recovered

The 110 AGW specimens of the 11 libraries generated a total of 6,509,970 sequence reads (Table 1). Clean reads were *de novo* assembled within each barcode and compared to the GenBank nonredundant protein database using BLASTx. Results indicated that HPV was the predominant virus detected in all the 11 libraries. Besides HPV, five libraries (GW02, GW06, GW07, GW09 and GW10) also yielded a small number of reads with sequence similarity to anellovirus which has been reported to be common in many tissues and bodily fluids [20]. Table 1 displayed the number of HPV sequence reads and the percentage of HPV reads against the total number of sequence reads in each library, which indicated that the percentage of HPV sequences in the 11 libraries ranged from 0.54% to 26.77% per library.

**Table 1.** Information of libraries and anogenital wart sample pools in viral metagenomic analysis.

Library ID	Sample pools composition	Total read no.	Read no. of HPV	Percentage of HPV reads (%)
GW01	No.61-70	85092	458	0.54
GW02	No.1-10	1300146	2746	0.21
GW03	No.11-110	97122	2865	2.65
GW04	No.71-80	416346	5471	1.31
GW05	No.21-30	599034	160352	26.77
GW06	No.51-60	244016	6496	2.66
GW07	No.31-40	831020	149524	17.99
GW08	No.11-20	1006036	6482	0.67
GW09	No.41-50	1133186	227374	20.07
GW10	No.91-100	312086	38302	12.27
GW11	No.81-90	485886	126407	26.02

### Read distribution of different types of HPV

**Figure 1.**
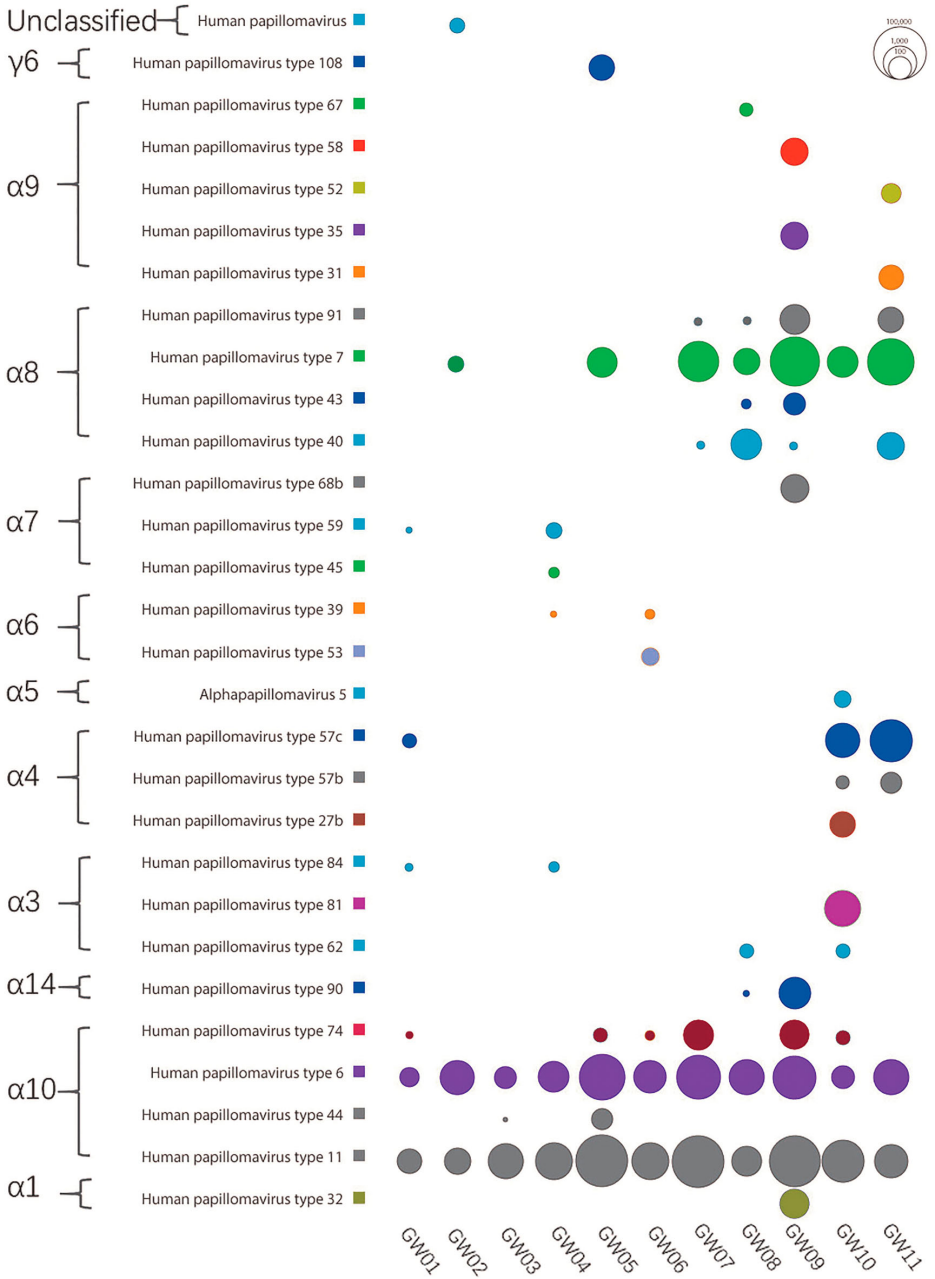
Sequence read distribution of different type HPVs revealed by viral metagenomics. The counts of sequence reads aligning to different types of HPV were calculated and represented using bubbles. Each bubble represents the log 10 transformed number of HPV reads. Species and type of HPVs detected in these libraries were labelled at left side and library IDs were labelled under the corresponding column.

BLASTx results generated by using DIAMOND were further analysed in MEGAN program, which yielded the HPV types read distribution in the 11 libraries. Short reads aligning to different types of HPV were counted and their log 10 transformed values were represented using bubbles (Figure 1). HPVs belonging to 11 different species, with 10 of them belonging in genus *Alphapapillomavirus* and one belonging in genus *Gammapapillomavirus*, and one unclassified type were detected. A total of 29 different HPV types were detected in the 11 sample pools. Over species level, *Alphapapillomavirus 10* and *Alphapapillomavirus 8* were the two dominant species detected in these libraries. Over HPV type level, HPV11 and HPV6 were detected in all 11 libraries. Besides the two common AGW-causing types (HPV11 and HPV6), HPV7 sequences were detected in 7 out of the 11 libraries, with 6 of them containing abundant HPV7 reads. In two libraries (i.e. GW09 and GW11) HPV7 belonged to the dominant type. HPV57c sequences were also detected with large number of sequence reads in three libraries (GW01, GW10 and GW11). High level of HPV40 sequence reads were present in two libraries (GW08 and GW11). A small number of HPV74 reads were detected in 6 libraries. Viral reads belonging to type HPV108 in species *Gammapapillomavirus 6* were present in one library (GW08). Besides, a small number of reads belonging to unclassified HPV were also detected in one library (GW01). HPV type specific read counts in the 11 libraries were presented Table S1.

### Complete genomes of HPV from the viral metagenomics and phylogenetic analysis

**Figure 2.**
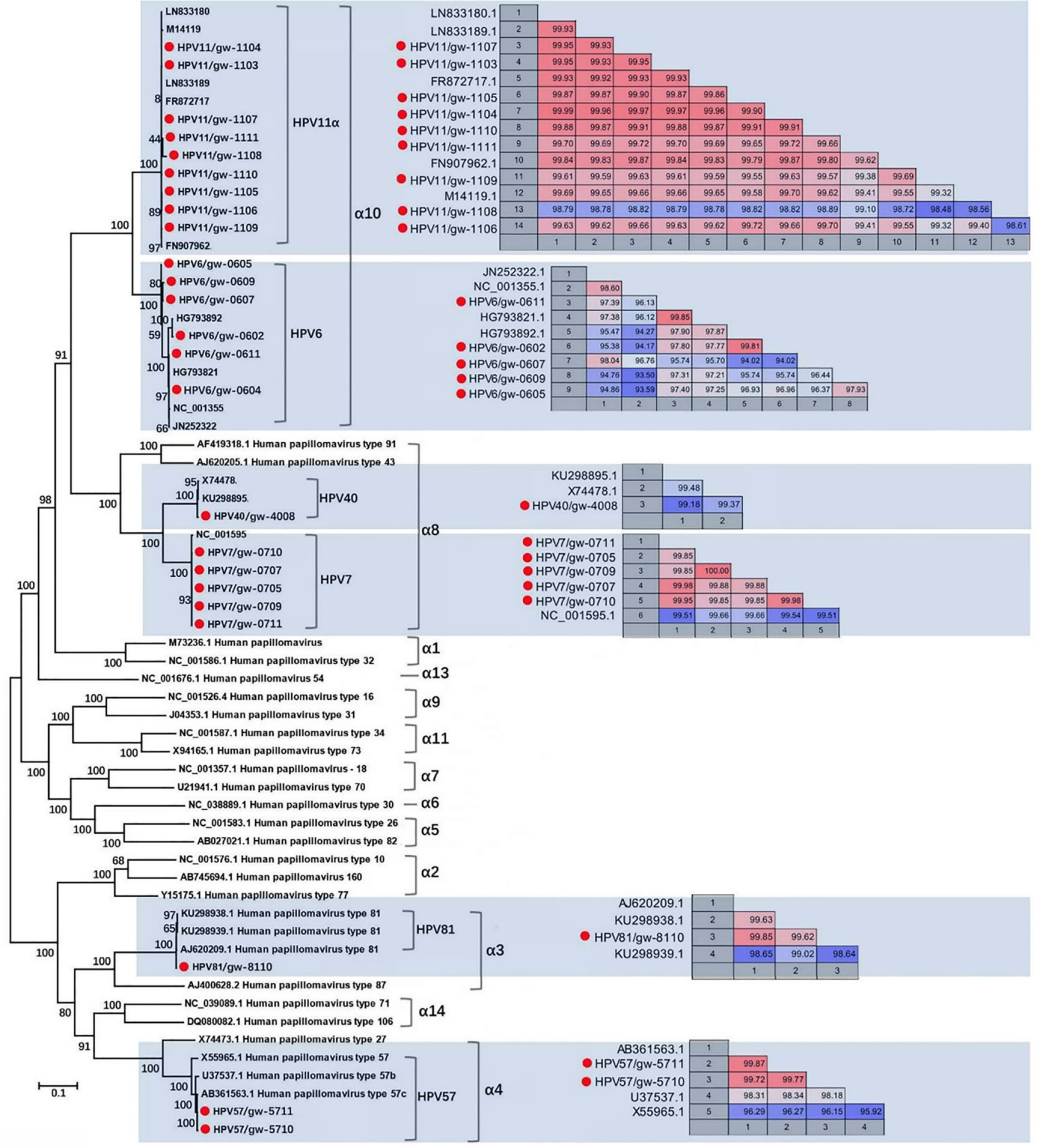
Phylogenetic analysis and pairwise sequence comparison based on the complete genome of HPV. The complete genomes of HPV in the phylogenetic analysis included the 24 complete genomes in this study, their closest relatives based on the BLASTn in GenBank, and other representative types of HPV in genus *Alphapapillomavirus*. The pairwise comparison based on complete genome among different strains within the same type of HPV was shown beside the corresponding clusters. The complete genomes acquired in this study were labelled with red dots.

Twenty-four HPV complete genomes were generated by *de novo* assembly, including 9 HPV11, 6 HPV6, 5 HPV7, 2 HPV57c, 1 HPV40, and 1 HPV81. We included HPV type and library ID from which the genome was acquired in the strain names of these 24 HPVs (Figure 2), respectively, which were then subjected to phylogenetic analysis based on the complete genome sequence. The pairwise nucleotide identities between different strains in the same type were shown beside the corresponding cluster (Figure 2). Based on complete genome sequence, the 9 HPV11 strains shared 98.56%-99.99% sequence similarities among themselves and their closest relatives. The 6 complete genomes of HPV6 were slightly more variable, sharing 94.02–99.85% sequence similarities among themselves and their closest relatives. In the α8 group, the genome of HPV40 showed 99.18% and 99.31% sequence similarities to the two complete genome sequences available in GenBank, respectively. The 5 complete genomes of HPV7 identified in this study shared 99.51–99.66% to the only complete HPV7 genome in GenBank (NC_001595) [21,22] and 99.85–100% among themselves with the strains from library GW05 and GW09 being identical. The complete genome of HPV81 from library GW10 showed 98.64%-99.85% similarities to the three complete genomes of HPV81 available in GenBank. The two HPV57 strains were closely clustered with HPV57c, sharing 99.72–99.87% sequence similarities to HPV57c strains available in GenBank.

### Prevalence of HPV7 in individual AGW specimens

Our viral metagenomic analysis revealed that HPV7 was present in 7 out of the 11 specimen pools, indicating that HPV7 was common in AGWs. In order to further test the prevalence of HPV7 in AGWs, two sets of nested-primers were designed based on the five complete genomes of HPV7 identified in this study and the other HPV7 strain available in GenBank. Nested PCR screening was then performed for another 190 individual AGW specimens (Table S2). PCR primers targeting L1 gene was used first. The specimens positive for L1 gene were then confirmed by PCR screening with the primers targeting E1 gene. Only the specimens showing positive in both PCRs were considered positive and the L1 gene fragments were Sanger sequenced. 25 out of the 190 (13.16%) AGW specimens were positive for HPV7. BLASTn searching based on the sequenced 357 bp L1 gene fragments confirmed that all of the 25 sequences belonged to HPV7, where 24 sequences of them were identical and had a nucleotide synonymous mutation compared to the rest one.

### Clinical and histological features of the HPV7-positive AGWs

**Figure 3.**
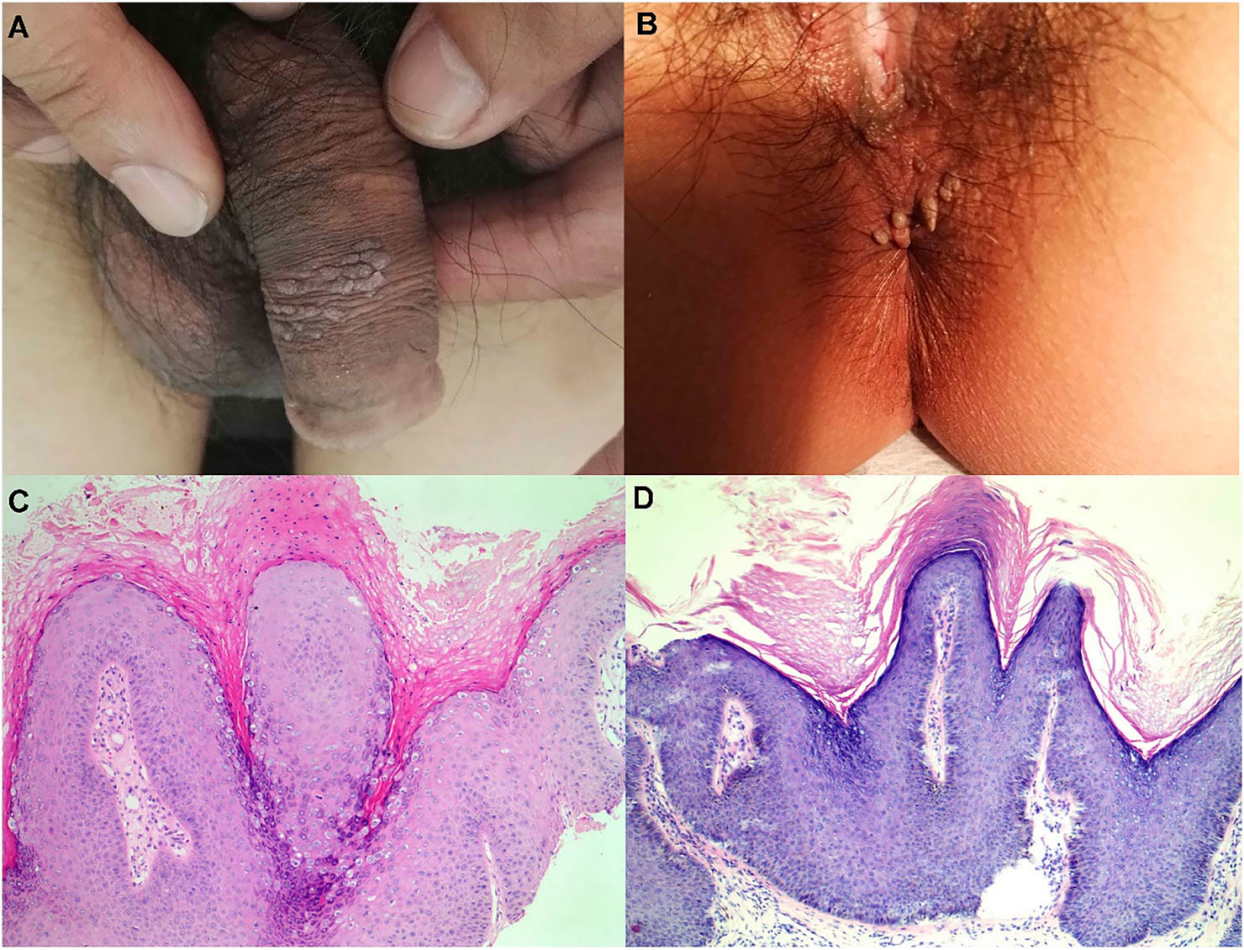
Representative lesions on the genital and haematoxylin and eosin staining results. (A) and (B): The two typical clinical presentation of these cases studied in this study. (C) and (D): Two representative histological findings of the two cases in (A) and (B), respectively, original magnification ×100.

The information of the 25 patients positive for HPV7 was presented in Table S2, of which 20 were male and 5 two were female. The mean age of the 25 patients was 48.04 ± 18.47(22-88) years, which is significantly older than those of non-HPV7-positive cases (*P* = .026). The average course of disease of the 25 HPV7-positive cases were 6.77 ± 4.74 months (1-24 months), which is significantly longer than those of non-HPV7-positive cases (*P *= .000). Figure 3(A,B) shows the two typical clinical presentation of these cases. All 25 HPV7 positive specimens were stained by haematoxylin and eosin. Figure 3(C,D) shows the two representative histological findings of the two cases in Figure 3(A,B), respectively, which indicated hyperkeratosis, parakeratosis, acanthosis, papillomatosis, and characteristic vacuolated parakeratotic and granular cells (koilocytes), being characteristics of HPV infection [23].

## Discussion

So far, over 200 types of HPV have been fully characterized and novel types of HPV are still being found [2,3,24–28]. Here, using viral metagenomics we described the profile of HPV types in 110 anogenital wart specimens which revealed the presence of 29 different HPV types. HPV11 and HPV6, two HPV types commonly causing anogenital warts, dominated in terms of the number of infected pools and read numbers. HPV7 were also detected with a high abundance of sequence reads, suggesting HPV7 might also be associated with AGW. Our molecular epidemiologic study based on another 190 individual AGW specimens using conventional PCR showed 13.16% (25/190) were positive for HPV7, suggesting HPV7 is prevalent in AGWs with high prevalence rate.

HPV 7 was initially discovered in hand warts of butchers and fish handlers [22,29,30] and subsequently detected in oral papillomas and face warts in HIV infected patients [31,32]. By southern blotting, HPV-7 DNA was also identified in two cases of condyloma [23]. Recently, HPV7 was also found in warts in toe webs (WTW) [33,34], suggested HPV7 might be associated with WTW. In this study, a high proportion of AGWs was found with HPV7 (13.16%) indicating a possible association with this condition. Five complete genomes of HPV7 were also acquired increasing the available number of HPV7 genomes from the single genome previously available.

AGWs are a common affliction, in which HPV6 and HPV11 are the most frequently detected papillomavirus types [35]. Factors associated with the incidence of AGW in men include younger age and high lifetime number of sexual partners [36]. AGWs treatment is expensive and often ineffective. Following AGW clearance with therapy, recurrence is common and is often seen within 3 months in 25% of cases, although rates of up to 67% have been observed [37,38]. Thus, developing effective strategies to prevent and control HPV infection is the most important way to lighten the burden of AGWs. Prophylactic HPV vaccines targeting the types likely to cause HPV-related cancer have been licensed for use, including a bivalent (HPV16, 18), quadrivalent (HPV6, 11, 16, 18) and nonavalent (HPV6, 11, 16, 18, 31, 33, 45, 52, 58) vaccines [39–41], of which the latter two contain HPV6 and HPV11. The role of HPV7 in AGW should be further investigated in order to better assess the need for its inclusion in future HPV vaccines.

In conclusion, our observations reinforce the view that HPV types of the HPV6/HPV11 group are the principal papillomaviruses associated with AGWs. It is of interest that HPV7, a type generally found in hand warts and recently in warts in toe webs, was also detected by viral metagenomics and conventional PCR in AGWs with high prevalence rate. Our data suggest that HPV7 should be considered in the future clinical test and vaccine development for anogenital warts. Further investigation based on a larger sample size of AGWs from other regions in China needs to be performed to elucidate the prevalence status of HPV7 in AGWs in China.

## Supplementary Material

Supplemental Material

## Data Availability

The 11 sets of raw sequence reads from the 11 viral metagenomic libraries were deposited in the Short Read Archive of GenBank database with accession no. SRX5313516- SRX5313526. The complete genome sequences human papillomavirus characterized in this study were submitted to GenBank with the accession no. MK463904-MK463926.
