# Supplementary material for: Viral metagenomics updated the prevalence of human papillomavirus types in anogenital warts
Source: Emerg Microbes Infect. 2019 Sep 7;8(1):1291–9. doi: 10.1080/22221751.2019.1661757 (PMC6746260; doi:10.1080/22221751.2019.1661757)
Supplement: Supplemental Material [file TEMI_A_1661757_SM8390.docx]

**Table S1.** Information about these patients whose AGW samples were in included viral metagenomic analysis.

| Sample No. | Gender | Age | Smapling time | Course(Months） | Location of wart* | Library ID | HPV type (specific read counts) |
| --- | --- | --- | --- | --- | --- | --- | --- |
| 1 | Male | 77 | 2016/9/20 | 3 | BS | GW02 | HPV6(2266)  HPV7(9)  HPV11(439)  Unclassified HPV (32) |
| 2 | Female | 38 | 2016/9/21 | 5 | CV | GW02 |  |
| 3 | Male | 79 | 2016/9/25 | 1 | PA | GW02 |  |
| 4 | Male | 46 | 2016/9/29 | 1 | FS,BS | GW02 |  |
| 5 | Male | 32 | 2016/10/2 | 2 | PA | GW02 |  |
| 6 | Male | 34 | 2016/10/7 | 0.5 | FS | GW02 |  |
| 7 | Male | 50 | 2016/10/9 | 2 | PA | GW02 |  |
| 8 | Male | 46 | 2016/10/10 | 2 | PA | GW02 |  |
| 9 | Female | 53 | 2016/10/11 | 5 | VV | GW02 |  |
| 10 | Male | 56 | 2016/10/11 | 2 | PA | GW02 |  |
| 11 | Female | 39 | 2016/10/14 | 1 | PM | GW08 | HPV6(3662)  HPV7(446)  HPV11(1227)  HPV40(1019)  HPV43(12)  HPV62(29)  HPV67(27)  HPV74(37)  HPV90(4)  HPV91(59) |
| 12 | Male | 47 | 2016/10/21 | 6 | GN | GW08 |  |
| 13 | Male | 54 | 2016/10/27 | 6 | MB,PA | GW08 |  |
| 14 | Male | 43 | 2016/10/28 | 3 | BS | GW08 |  |
| 15 | Male | 16 | 2016/10/28 | 2 | PA | GW08 |  |
| 16 | Female | 44 | 2016/10/28 | 2 | VV,PA | GW08 |  |
| 17 | Female | 56 | 2016/10/29 | 3 | VV,PA | GW08 |  |
| 18 | Female | 40 | 2016/10/31 | 3 | PA | GW08 |  |
| 19 | Male | 54 | 2016/11/3 | 12 | FS,BS | GW08 |  |
| 20 | Female | 32 | 2016/11/4 | 6 | VN | GW08 |  |
| 21 | Female | 56 | 2016/11/6 | 2 | VV | GW05 | HPV6(28233)  HPV7(835)  HPV11(129925)  HPV44(119)  HPV74(877)  HPV108(363) |
| 22 | Male | 50 | 2016/11/8 | 0.6 | BS | GW05 |  |
| 23 | Male | 48 | 2016/11/17 | 3 | PA | GW05 |  |
| 24 | Male | 35 | 2016/11/19 | 2 | FS | GW05 |  |
| 25 | Male | 48 | 2016/11/22 | 2 | BS,PA | GW05 |  |
| 26 | Male | 32 | 2016/11/25 | 12 | PB | GW05 |  |
| 27 | Male | 32 | 2016/11/28 | 1 | BS,MB | GW05 |  |
| 28 | Male | 84 | 2016/12/4 | 6 | BS | GW05 |  |
| 29 | Male | 29 | 2016/12/8 | 2 | MB,PA | GW05 |  |
| 30 | Female | 28 | 2016/12/9 | 2 | VV | GW05 |  |
| 31 | Female | 50 | 2016/12/14 | 4 | PM | GW07 | HPV6(22001)  HPV7(10221)  HPV11(116026)  HPV40(273)  HPV74 (862)  HPV91(141) |
| 32 | Male | 51 | 2016/12/16 | 2 | PM | GW07 |  |
| 33 | Female | 54 | 2016/12/19 | 12 | VN | GW07 |  |
| 34 | Male | 40 | 2016/12/23 | 3 | FS | GW07 |  |
| 35 | Male | 27 | 2017/1/4 | 6 | FS,BS | GW07 |  |
| 36 | Male | 37 | 2017/1/11 | 5 | US | GW07 |  |
| 37 | Male | 70 | 2017/1/16 | 2 | PA | GW07 |  |
| 38 | Male | 34 | 2017/1/20 | 15 | PA | GW07 |  |
| 39 | Male | 27 | 2017/1/21 | 0.3 | BS | GW07 |  |
| 40 | Male | 71 | 2017/2/4 | 1 | GN | GW07 |  |
| 41 | Male | 23 | 2017/2/6 | 2 | PA | GW09 | HPV6(20115)  HPV7(86212)  HPV11(112521)  HPV32(894)  HPV35(672)  HPV40(1365)  HPV43(181)  HPV58(587)  HPV68b(912)  HPV74(971)  HPV90(1712)  HPV91(1232) |
| 42 | Female | 38 | 2017/2/9 | 0.5 | VV | GW09 |  |
| 43 | Male | 45 | 2017/2/10 | 2 | PA | GW09 |  |
| 44 | Female | 54 | 2017/2/14 | 1 | VV | GW09 |  |
| 45 | Male | 24 | 2017/2/15 | 0.25 | FS | GW09 |  |
| 46 | Male | 26 | 2017/2/16 | 1 | BS | GW09 |  |
| 47 | Male | 24 | 2017/2/18 | 6 | FS,BS | GW09 |  |
| 48 | Male | 43 | 2017/2/18 | 1 | PA | GW09 |  |
| 49 | Male | 31 | 2017/2/21 | 2 | FS,BS | GW09 |  |
| 50 | Male | 74 | 2017/3/3 | 2 | PA | GW09 |  |
| 51 | Male | 42 | 2017/3/5 | 3 | PA | GW06 | HPV6(1858)  HPV11(4449)  HPV39(10)  HPV53(60)  HPV74(119) |
| 52 | Male | 62 | 2017/3/6 | 5 | FS,BS | GW06 |  |
| 53 | Female | 37 | 2017/3/7 | 3 | PA | GW06 |  |
| 54 | Female | 31 | 2017/3/14 | 5 | PA | GW06 |  |
| 55 | Male | 34 | 2017/3/14 | 1 | BS | GW06 |  |
| 56 | Male | 54 | 2017/3/17 | 1 | BS | GW06 |  |
| 57 | Male | 23 | 2017/3/22 | 1 | FS,BS | GW06 |  |
| 58 | Male | 22 | 2017/3/23 | 0.5 | FS,BS | GW06 |  |
| 59 | Female | 28 | 2017/3/25 | 1 | VV | GW06 |  |
| 60 | Male | 35 | 2017/3/29 | 3 | PA | GW06 |  |
| 61 | Male | 30 | 2017/3/29 | 0.667 | BS | GW01 | HPV6(94)  HPV11(319)  HPV57c(28)  HPV59(5)  HPV74(6)  HPV84(6) |
| 62 | Female | 32 | 2017/3/31 | 2 | PM | GW01 |  |
| 63 | Female | 26 | 2017/4/4 | 1 | VN | GW01 |  |
| 64 | Male | 36 | 2017/4/6 | 2 | PA | GW01 |  |
| 65 | Female | 29 | 2017/4/10 | 2 | VV | GW01 |  |
| 66 | Female | 27 | 2017/4/12 | 2 | VV | GW01 |  |
| 67 | Female | 51 | 2017/4/13 | 2 | PA | GW01 |  |
| 68 | Male | 50 | 2017/4/15 | 1 | BS | GW01 |  |
| 69 | Female | 40 | 2017/4/21 | 5 | VV | GW01 |  |
| 70 | Male | 25 | 2017/4/23 | 2 | MB,PA | GW01 |  |
| 71 | Male | 32 | 2017/4/27 | 0.25 | FS | GW04 | HPV6(1344)  HPV11(4036)  HPV39(7)  HPV45(17)  HPV59 (48)  HPV84(19) |
| 72 | Male | 26 | 2017/4/28 | 6 | PM | GW04 |  |
| 73 | Male | 53 | 2017/4/29 | 1 | FS,BS | GW04 |  |
| 74 | Male | 70 | 2017/5/2 | 0.25 | US | GW04 |  |
| 75 | Male | 37 | 2017/5/3 | 2 | PA | GW04 |  |
| 76 | Male | 71 | 2017/5/5 | 12 | FS,BS,PA | GW04 |  |
| 77 | Female | 54 | 2017/5/6 | 3 | PM | GW04 |  |
| 78 | Female | 50 | 2017/5/7 | 2 | VV | GW04 |  |
| 79 | Male | 28 | 2017/5/12 | 6 | PM | GW04 |  |
| 80 | Male | 27 | 2017/5/16 | 6 | PM | GW04 |  |
| 81 | Male | 40 | 2017/5/22 | 4 | PA | GW11 | HPV6(6542)  HPV7(79981)  HPV11(4025)  HPV31(621)  HPV40(1110)  HPV52(191)  HPV57b(271)  HPV57c(32912)  HPV91(754) |
| 82 | Female | 25 | 2017/5/24 | 2 | CV | GW11 |  |
| 83 | Female | 27 | 2017/5/24 | 1 | PA | GW11 |  |
| 84 | Male | 56 | 2017/5/28 | 1 | FS,BS | GW11 |  |
| 85 | Male | 63 | 2017/5/31 | 1 | PA | GW11 |  |
| 86 | Male | 46 | 2017/6/7 | 2 | FS,BS | GW11 |  |
| 87 | Female | 41 | 2017/6/9 | 2 | VV,PA | GW11 |  |
| 88 | Male | 68 | 2017/6/12 | 1 | BS | GW11 |  |
| 89 | Male | 46 | 2017/6/12 | 2 | FS,BS | GW11 |  |
| 90 | Male | 55 | 2017/6/12 | 5 | FS | GW11 |  |
| 91 | Female | 32 | 2017/6/13 | 6 | VV | GW10 | HPV6(301)  HPV7(2013)  HPV11(24476)  HPV27b(645)  HPV57b(38)  HPV57c(4260)  HPV62(43)  HPV74(162)  HPV81(6287)  Alphapapillomavirus 5(77) |
| 92 | Male | 62 | 2017/6/13 | 6 | FS,BS | GW10 |  |
| 93 | Male | 46 | 2017/6/13 | 2 | PA | GW10 |  |
| 94 | Male | 37 | 2017/6/19 | 12 | PB | GW10 |  |
| 95 | Female | 57 | 2017/6/27 | 1 | PM | GW10 |  |
| 96 | Male | 24 | 2017/6/28 | 1 | FS,BS,PA | GW10 |  |
| 97 | Male | 42 | 2017/6/30 | 2 | FS | GW10 |  |
| 98 | Female | 49 | 2017/7/4 | 1 | VN | GW10 |  |
| 99 | Male | 55 | 2017/7/5 | 2 | FS | GW10 |  |
| 100 | Female | 32 | 2017/7/5 | 2 | PA | GW10 |  |
| 101 | Female | 30 | 2017/7/18 | 6 | VV | GW03 | HPV6(137)  HPV11(2722)  HPV44(6) |
| 102 | Male | 35 | 2017/7/18 | 6 | PM | GW03 |  |
| 103 | Male | 50 | 2017/8/1 | 5 | FS,BS | GW03 |  |
| 104 | Male | 32 | 2017/8/3 | 12 | PB | GW03 |  |
| 105 | Male | 32 | 2017/8/4 | 1 | FS | GW03 |  |
| 106 | Male | 56 | 2017/8/5 | 6 | FS,BS | GW03 |  |
| 107 | Male | 62 | 2017/8/5 | 0.25 | FS,BS | GW03 |  |
| 108 | Male | 25 | 2017/8/5 | 13 | PA | GW03 |  |
| 109 | Female | 26 | 2017/8/7 | 2 | VV | GW03 |  |
| 110 | Female | 44 | 2017/8/15 | 2 | CV | GW03 |  |

*: Balanus=BS, Penis body=PB, Foreskin=FS, Vulva=VV, Perianal=PA, Mons pubis=MB, Urethral orifics=US, Vagina=VN, Cervix=CV, Groin=GN, Perineum=PM

**Table S2.** Information about these patients whose AGW samples were in included in the conventional PCR screening for HPV7.

| **Sample No.** | **Gender** | **Age** | **Course(months)** | **Location of wart#** | **Sampling time** |
| --- | --- | --- | --- | --- | --- |
| 1 | Female | 24 | 4 | VV,PA | 2017/8/20 |
| 2 | Female | 44 | 5 | VV | 2017/8/25 |
| 3 | Male | 33 | 6 | PM,US | 2017/8/28 |
| 4* | Male | 58 | 6 | BS | 2017/8/29 |
| 5 | Female | 48 | 2 | PA | 2017/8/30 |
| 6* | Male | 23 | 12 | PB | 2017/9/1 |
| 7 | Female | 21 | 1 | PM | 2017/9/1 |
| 8* | Female | 22 | 6 | PM | 2017/9/1 |
| 9 | Male | 46 | 5 | PA,FS | 2017/9/6 |
| 10 | Male | 38 | 2 | FS | 2017/9/19 |
| 11 | Male | 54 | 1 | FS,BS | 2017/9/20 |
| 12 | Male | 66 | 2 | MB,PA | 2017/9/21 |
| 13 | Male | 28 | 2 | PA | 2017/9/27 |
| 14 | Female | 52 | 1 | VN | 2017/9/28 |
| 15 | Male | 33 | 6 | PM | 2017/9/28 |
| 16 | Male | 29 | 2 | PA | 2017/10/10 |
| 17* | Male | 42 | 6 | PB | 2017/10/13 |
| 18 | Male | 44 | 1 | PM | 2017/10/16 |
| 19 | Male | 40 | 6 | FS,BS | 2017/10/17 |
| 20 | Male | 37 | 1 | FS | 2017/10/25 |
| 21 | Male | 32 | 6 | FS,BS,PA | 2017/10/26 |
| 22 | Male | 33 | 1 | FS | 2017/11/3 |
| 23 | Male | 25 | 1 | GN | 2017/11/6 |
| 24 | Female | 32 | 5 | VV | 2017/11/14 |
| 25 | Female | 65 | 4 | CV | 2017/11/14 |
| 26 | Male | 62 | 1 | FS,BS | 2017/11/15 |
| 27 | Male | 34 | 7 | FS | 2017/11/20 |
| 28 | Male | 43 | 2 | MB | 2017/11/22 |
| 29 | Female | 66 | 12 | VV,PA | 2017/11/24 |
| 30 | Female | 25 | 2 | PA | 2017/11/25 |
| 31 | Male | 48 | 1 | BS | 2017/12/2 |
| 32 | Female | 23 | 5 | VV | 2017/12/4 |
| 33 | Male | 62 | 6 | PM | 2017/12/5 |
| 34 | Male | 37 | 7 | BS | 2017/12/11 |
| 35* | Male | 27 | 6 | FS,BS,PA | 2017/12/18 |
| 36 | Male | 77 | 3 | FS,BS,PA | 2017/12/18 |
| 37 | Female | 43 | 2 | PA | 2017/12/19 |
| 38 | Male | 33 | 5 | FS,BS | 2017/12/23 |
| 39 | Male | 38 | 1 | PA | 2017/12/26 |
| 40 | Female | 23 | 3 | VV | 2017/12/27 |
| 41 | Female | 26 | 1 | PA | 2018/1/2 |
| 42 | Male | 29 | 15 | MB | 2018/1/2 |
| 43 | Male | 54 | 2 | FS,BS | 2018/1/4 |
| 44 | Male | 41 | 4 | US | 2018/1/8 |
| 45 | Male | 34 | 1 | MB | 2018/1/8 |
| 46 | Male | 34 | 2 | FS | 2018/1/8 |
| 47 | Male | 23 | 2 | MB | 2018/1/8 |
| 48 | Male | 28 | 2 | MB,PA | 2018/1/8 |
| 49 | Male | 41 | 2 | PA | 2018/1/10 |
| 50 | Male | 37 | 1 | BS | 2018/1/12 |
| 51 | Male | 27 | 5 | FS | 2018/1/13 |
| 52 | Female | 32 | 6 | PM | 2018/1/15 |
| 53 | Male | 22 | 6 | BS | 2018/1/19 |
| 54 | Male | 34 | 2 | PA | 2018/1/22 |
| 55 | Male | 55 | 2 | PB | 2018/1/22 |
| 56 | Male | 30 | 1 | FS,BS,PA | 2018/1/24 |
| 57 | Male | 51 | 1 | PA | 2018/1/29 |
| 58 | Female | 29 | 3 | VV | 2018/2/7 |
| 59 | Male | 36 | 3 | PA | 2018/2/7 |
| 60 | Female | 47 | 1 | VV | 2018/2/9 |
| 61 | Male | 42 | 7 | FS,BS | 2018/2/12 |
| 62 | Male | 32 | 2 | MB | 2018/2/24 |
| 63 | Female | 64 | 4 | VV,PA | 2018/2/26 |
| 64 | Male | 24 | 2 | PA,FS | 2018/2/27 |
| 65 | Female | 46 | 1 | VN | 2018/3/1 |
| 66 | Male | 37 | 5 | GN,PB | 2018/3/1 |
| 67 | Female | 59 | 6 | CV | 2018/3/5 |
| 68 | Male | 32 | 4 | BS | 2018/3/6 |
| 69 | Male | 24 | 12 | FS,BS,PA | 2018/3/9 |
| 70 | Female | 56 | 1 | CV | 2018/3/10 |
| 71* | Male | 74 | 6 | GN | 2018/3/12 |
| 72* | Male | 60 | 1 | MB,FS | 2018/3/15 |
| 73 | Male | 47 | 3 | PM | 2018/3/16 |
| 74 | Female | 26 | 4 | VV | 2018/3/20 |
| 75 | Male | 74 | 1 | FS | 2018/3/23 |
| 76 | Male | 23 | 1 | PA | 2018/3/23 |
| 77 | Male | 24 | 1 | FS,BS | 2018/3/29 |
| 78* | Male | 28 | 12 | PA | 2018/4/2 |
| 79 | Male | 33 | 1 | FS,BS | 2018/4/3 |
| 80 | Male | 54 | 2 | FS | 2018/4/4 |
| 81 | Male | 46 | 2 | FS,BS | 2018/4/4 |
| 82 | Female | 32 | 2 | VV,PA | 2018/4/5 |
| 83 | Male | 39 | 2 | US | 2018/4/6 |
| 84 | Male | 63 | 1 | BS | 2018/4/6 |
| 85 | Female | 80 | 5 | FS | 2018/4/7 |
| 86 | Male | 45 | 2 | PA | 2018/4/13 |
| 87 | Male | 26 | 6 | PB | 2018/4/16 |
| 88 | Female | 35 | 1 | PM | 2018/4/18 |
| 89 | Male | 27 | 1 | PA | 2018/4/19 |
| 90 | Male | 37 | 2 | MB | 2018/4/19 |
| 91 | Male | 44 | 3 | MB | 2018/4/19 |
| 92 | Male | 37 | 2 | PA,MB | 2018/4/20 |
| 93 | Male | 55 | 2 | FS,PA | 2018/4/23 |
| 94 | Female | 37 | 1 | PA | 2018/4/23 |
| 95 | Male | 23 | 6 | MB,FS | 2018/4/30 |
| 96 | Male | 24 | 1 | PA | 2018/4/30 |
| 97 | Male | 44 | 1 | FS | 2018/5/3 |
| 98 | Female | 29 | 4 | MB | 2018/5/5 |
| 99* | Male | 25 | 24 | GN | 2018/5/8 |
| 100 | Female | 33 | 1 | VV | 2018/5/14 |
| 101 | Male | 35 | 2 | FS | 2018/5/14 |
| 102 | Male | 47 | 2 | FS | 2018/5/15 |
| 103 | Male | 57 | 2 | BS,PA | 2018/5/16 |
| 104 | Male | 52 | 2 | PA | 2018/5/16 |
| 105 | Male | 51 | 1 | BS | 2018/5/17 |
| 106 | Male | 58 | 5 | FS | 2018/5/18 |
| 107 | Male | 35 | 6 | PM | 2018/5/21 |
| 108 | Female | 35 | 6 | VV | 2018/5/22 |
| 109 | Female | 38 | 2 | CV | 2018/5/23 |
| 110 | Male | 36 | 4 | PB | 2018/5/24 |
| 111 | Male | 44 | 1 | PM | 2018/5/24 |
| 112 | Male | 29 | 6 | BS | 2018/5/25 |
| 113 | Male | 33 | 2 | FS | 2018/5/25 |
| 114 | Male | 26 | 3 | PB | 2018/5/29 |
| 115 | Male | 40 | 1 | PM | 2018/5/31 |
| 116 | Male | 36 | 1 | PA | 2018/6/1 |
| 117 | Female | 26 | 2 | VV | 2018/6/4 |
| 118* | Female | 43 | 2 | PA | 2018/6/5 |
| 119 | Male | 25 | 1 | FS | 2018/6/5 |
| 120* | Male | 53 | 7 | MB | 2018/6/6 |
| 121 | Male | 47 | 2 | MB | 2018/6/13 |
| 122 | Male | 30 | 4 | GN,PA | 2018/6/16 |
| 123 | Male | 33 | 2 | PA | 2018/6/19 |
| 124 | Male | 42 | 1 | FS | 2018/6/19 |
| 125 | Male | 40 | 5 | MB | 2018/6/21 |
| 126 | Male | 28 | 6 | PM | 2018/6/22 |
| 127 | Male | 24 | 7 | BS | 2018/6/24 |
| 128 | Male | 44 | 5 | MB | 2018/6/25 |
| 129 | Male | 64 | 1 | PA | 2018/7/2 |
| 130 | Female | 31 | 4 | VV | 2018/7/3 |
| 131 | Male | 27 | 1 | MB | 2018/7/4 |
| 132 | Male | 27 | 1 | BS | 2018/7/4 |
| 133 | Male | 42 | 3 | PM | 2018/7/4 |
| 134 | Male | 42 | 3 | BS | 2018/7/4 |
| 135* | Male | 51 | 3 | MB | 2018/7/5 |
| 136 | Male | 51 | 1 | BS | 2018/7/5 |
| 137 | Male | 32 | 1 | BS | 2018/7/6 |
| 138 | Male | 32 | 2 | FS,PB | 2018/7/6 |
| 139* | Male | 35 | 5 | PA | 2018/7/9 |
| 140 | Male | 53 | 5 | MB | 2018/7/9 |
| 141 | Male | 58 | 1 | FS | 2018/7/11 |
| 142 | Female | 18 | 3 | PA | 2018/7/11 |
| 143 | Male | 40 | 1 | BS | 2018/7/12 |
| 144 | Male | 37 | 2 | BS | 2018/7/12 |
| 145 | Male | 37 | 2 | US,PA | 2018/7/13 |
| 146 | Female | 23 | 3 | PA | 2018/7/13 |
| 147 | Male | 38 | 4 | PA | 2018/7/14 |
| 148 | Male | 29 | 2 | PB | 2018/7/16 |
| 149 | Female | 48 | 1 | VV | 2019/1/29 |
| 150* | Male | 53 | 3 | BS,FS | 2019/2/2 |
| 151 | Male | 20 | 1 | FS | 2019/2/11 |
| 152 | Male | 43 | 2 | BS | 2019/2/15 |
| 153* | Female | 81 | 6 | VV,VN,PA | 2019/2/18 |
| 154 | Male | 35 | 2 | PA | 2019/2/19 |
| 155 | Female | 38 | 1 | VN | 2019/2/20 |
| 156 | Male | 26 | 2 | PA | 2019/2/20 |
| 157 | Male | 33 | 2 | FS | 2019/2/22 |
| 158 | Male | 33 | 1 | FS | 2019/2/25 |
| 159 | Male | 51 | 3 | PB | 2019/2/25 |
| 160 | Male | 36 | 2 | PA | 2019/2/26 |
| 161* | Male | 67 | 12 | BS,FS | 2019/3/4 |
| 162 | Female | 34 | 2 | VV,VN | 2019/3/7 |
| 163* | Female | 50 | 7 | PA | 2019/3/8 |
| 164* | Male | 55 | 8 | PB,PA | 2019/3/11 |
| 165 | Male | 27 | 2 | PB | 2019/3/11 |
| 166 | Male | 24 | 1 | PA | 2019/3/12 |
| 167 | Male | 29 | 2 | PB | 2019/3/12 |
| 168 | Female | 44 | 1 | VV,VN | 2019/3/13 |
| 169* | Male | 37 | 2 | PB | 2019/3/16 |
| 170 | Male | 74 | 2 | BS,FS | 2019/3/19 |
| 171 | Male | 42 | 4 | PB | 2019/3/22 |
| 172* | Male | 42 | 5 | BS | 2019/3/24 |
| 173 | Male | 25 | 2 | FS | 2019/3/26 |
| 174 | Male | 42 | 3 | US | 2019/3/29 |
| 175 | Female | 34 | 2 | VV | 2019/3/29 |
| 176* | Male | 43 | 4 | PA | 2019/3/30 |
| 177 | Female | 44 | 2 | VV,VN | 2019/3/31 |
| 178* | Male | 74 | 9 | VV | 2019/4/2 |
| 179 | Male | 38 | 3 | PB | 2019/4/2 |
| 180 | Male | 21 | 1 | BS | 2019/4/2 |
| 181 | Male | 30 | 2 | PB | 2019/4/9 |
| 182* | Female | 37 | 4 | VV,VN | 2019/4/9 |
| 183 | Male | 53 | 2 | FS | 2019/4/12 |
| 184 | Male | 26 | 2 | PB | 2019/4/15 |
| 185 | Male | 74 | 2 | BS,FS | 2019/4/15 |
| 186 | Male | 28 | 1 | US | 2019/4/15 |
| 187 | Male | 31 | 4 | FS | 2019/4/16 |
| 188* | Male | 88 | 11 | BS,FS | 2019/4/18 |
| 189 | Male | 55 | 3 | PA | 2019/4/18 |
| 190* | Male | 33 | 5 | GN | 2019/4/22 |

#: Balanus=BS, Penis body=PB, Mons pubis=MB, Foreskin=FS, Vulva=VV, Perianal=PA, Urethral orifics=US, Vagina=VN, Cervix=CV, Groin=GN, Perineum=PM

*: Sample positive for HPV7 in PCR screening.
